# Rise in Group W Meningococcal Carriage in University Students, United Kingdom

**DOI:** 10.3201/eid2306.161768

**Published:** 2017-06

**Authors:** Neil J. Oldfield, Caroline Cayrou, Mahab A.K. AlJannat, Ali A.A. Al-Rubaiawi, Luke R. Green, Shehzan Dada, Oliver D. Steels, Christopher Stirrup, Joe Wanford, Banan A.Y. Atwah, Christopher D. Bayliss, David P.J. Turner

**Affiliations:** University of Nottingham, Nottingham, UK (N.J. Oldfield, M.A.K. AlJannat, O.D. Steels, C. Stirrup, B.A.Y. Atwah, D.P.J. Turner);; University of Leicester, Leicester, UK (C. Cayrou, A.A.A. Al-Rubaiawi, L.R. Green, S. Dada, J. Wanford, C.D. Bayliss)

**Keywords:** Neisseria meningitidis, meningitis/encephalitis, carrier state, students, meningococcus, serogroup W-135, MenACWY, epidemiology, vaccine, bacteria, United Kingdom

## Abstract

MenACWY conjugate vaccination was recently introduced in the United Kingdom for adolescents and young adults to reduce disease from infection by *Neisseria meningitidis* group W. We conducted a cross-sectional meningococcal carriage study in first-year UK university students. Despite 71% MenACWY vaccine coverage, carriage of group W increased substantially.

*Neisseria meningitidis* causes severe sepsis and meningitis. The main reservoir in most populations is asymptomatic pharyngeal carriage in older adolescents and young adults (*1*). High carriage rates are particularly evident in semiclosed communities of young adults, such as university student populations, where persons live, work, and socialize together (*2*). 

Meningococcal carriage was previously assessed in university students in the United Kingdom during 2009–10 at the University of Nottingham (UoN) when a high prevalence of capsular group Y (MenY) meningococcal carriage was detected (*3*). This high level of MenY carriage was concomitant with a rise in disease caused by MenY strains in the United Kingdom (*4*). Since 2009, although MenY disease has plateaued, capsular group W (MenW) disease has steadily increased because of the rapid expansion of hypervirulent strains belonging to the sequence type 11 clonal complex (MenW:ST-11) (*5*). 

Analysis of whole-genome sequence data has shown that isolates from the same MenW:ST-11 lineage, termed the South American/UK strain, are also endemic to Chile, Brazil, and Argentina (*6*) and were recently reported in Australia (*7*). In response, Public Health England introduced the MenACWY vaccine in the routine adolescent school program for 14- and 15-year-olds and first-year university students (*8*). A catch-up campaign was used to offer MenACWY vaccine to all 14–18-year-old persons, with persons who left school in 2015 (17–18 years of age) given priority.

The rationale for targeting the vaccine to older adolescents and young adults stems from carriage studies showing that this demographic represents the principal reservoir of meningococcal carriage, as well as experience with other polysaccharide conjugate vaccines. Introduction of the MenC monovalent conjugate vaccines previously reduced carriage acquisition of MenC strains in adolescents and young adults (*9*). There is evidence, albeit limited, to suggest that the quadrivalent vaccine may have a similar effect on carriage of MenCWY strains (*10*,*11*). Reduced carriage in this population should lead to indirect protection of other age groups through herd immunity, thus enhancing the public health effect and cost-effectiveness of this approach.

## The Study

To assess trends in meningococcal strain carriage, and to determine the immediate effect of the MenACWY vaccine on carriage of MenW/Y strains, we conducted a cross-sectional study in first-year students at the UoN from registration during September 2015 through March 2016. MenACWY vaccination coverage in this student population increased from 31% (preregistration) to ≈70% (immediately postregistration) as a result of a campus-based vaccination campaign targeting freshmen (*12*). 

The study was approved by the Research Ethics Committee at the UoN, and written informed consent was obtained from all participants. We recruited convenience samples of first-year students in September and November 2015 and March 2016. In September, students were recruited during registration; in November and March, students were recruited in 5 dormitories (A–E) with single-occupancy rooms. We searched the UoN Health Service registration database (EMIS Web software; EMIS Health, Leeds, UK) to determine vaccination status in registered first-year students on arrival at the UoN and after the campus-based vaccination campaign.

In September, we obtained pharyngeal swab specimens from eligible students immediately before they received the MenACWY vaccine. We immediately inoculated all pharyngeal swabs onto GC selective agar (Oxoid, Basingstoke, UK) and incubated them at 37°C in air containing 5% CO_2_. After 24 and 48 hours, we selected oxidase-positive colonies suggestive of *Neisseria* spp. and confirmed their identity by amplification of the meningococcal gene *crgA* plus *ctrA* and/or *porA*, as described previously (*13*).

We performed PCR-based genogrouping as described previously (*13*,*14*). The Meningococcal Reference Unit, Public Health England, Manchester, UK, performed serogrouping and serotyping of MenW isolates using dot-blot enzyme immunoassay. Sequence data derived from amplified *porA* and factor H–binding protein alleles was queried against the PubMLST/Neisseria database (http://pubmlst.org/neisseria). We performed χ^2^ tests for significance by using STATCALC (Epi Info version 7.2.0.1; Centers for Disease Control and Prevention, Atlanta, GA, USA).

The September sample of 769 first-year students represented 10.9% of the 7,049 first-year students registered in 2015. Carriage rates among this group increased from 14% in late September 2015 to 39% by mid-November 2015 and 46% in March 2016 (Technical Appendix Table 1). The characteristics of enrolled students and behavioral risk factors for carriage were similar at the 3 time points. The initial carriage rate of 14% was much lower for first-year students in September 2015 than in September 2009 (23.2%; χ^2^ = 34, df = 1; p<0.00001) (*3*), suggesting a reduction in meningococcal carriage in adolescents in the United Kingdom, possibly attributable to an alteration in risk factors for carriage. The MenY carriage rate for incoming students (1.8%) was also lower than that detected in 2009 (2.9%; χ^2^ = 2.0, df = 1; p = 0.15) (*3*).

In September 2015, carriage rates were 4.2% for genogroup capsule null locus (cnl), 3.3% for B, 1.8% for Y, and 0.7% for W strains. A substantial part of the increase in carriage from September 2015 through March 2016 was the result of a notable increase (0.7% to 8.0%) in carriage of MenW strains (Technical Appendix Table 1). No change in the carriage of MenY strains was detected (Technical Appendix Table 1). Of the 50 students colonized with MenW, 36 (72%) had received MenACWY vaccine either before or during registration, which is consistent with the overall MenACWY coverage in our first-year student cohort. Students colonized with MenW at the second and third sampling times were distributed across all 5 dormitories, suggesting widespread dissemination, and 21 (91%) of the MenW carriers in the last time point (March 2016) had been vaccinated at least 5 months before sampling (Technical Appendix Table 2).

Analysis of the genogroup W isolates showed that 47 (90%) of 52 were serotype 2a (Technical Appendix Table 1) and harbored alleles for factor H binding protein peptide 22 and PorA P1.5,2, identical to the corresponding alleles harbored by the MenW:ST-11 clone responsible for the increase in invasive MenW disease in the United Kingdom (*5*). We examined capsular expression by serogrouping and found 32 (62%) of the MenW isolates expressed the W capsular polysaccharide. Of the 21 MenW carriers in March 2016 who had been vaccinated at least 5 months before sampling, 15 (71%) were harboring isolates expressing the W capsule (Technical Appendix Table 2).

## Conclusions

We detected a rapid rise in carriage of MenW among first-year students in a university setting in the United Kingdom. In comparison, carriage of MenC in adolescents and young adults in the United Kingdom, including in university students, was rare before the introduction of MenC monovalent conjugate vaccines (*9*). The rise in MenW carriage is most likely due to acquisition within student dormitories or social spaces on campus but was unexpected because no such isolates were found in a similar study at the UoN in 2008–09 (*15*), and only a limited number in a multicenter carriage study involving UK universities in 2010–11 (*6*,*10*).

The increase in MenW carriage among university students merits further monitoring because it could contribute further to the sustained increase in MenW disease in the United Kingdom. Two cases of MenW disease were reported in unvaccinated students in Nottingham during 2015–16. Students attending universities exhibit high mobility and may represent an ongoing vehicle for amplification and spread of MenW into communities throughout the United Kingdom or beyond, with implications for vaccination policy and future research.

Technical Appendix Characteristics of meningococcal carriage in first-year university students and MenW carriers in each of 5 dormitories and their respective MenACWY vaccination status, University of Nottingham, UK, 2015–16
