# Supplementary material for: Rise in Group W Meningococcal Carriage in University Students, United Kingdom
Source: Emerg Infect Dis. 2017 Jun;23(6):1009–11. doi: 10.3201/eid2306.161768 (PMC5443439; doi:10.3201/eid2306.161768)
Supplement: Technical Appendix — Characteristics of meningococcal carriage in first-year university students and MenW carriers in each of 5 dormitories and their respective MenACWY vaccination status, University of Nottingham, UK, 2015–16 [file 16-1768-Techapp-s1.pdf]

Article DOI: http://dx.doi.org/10.3201/eid2306.161768

Rise in Group W Meningococcal Carriage in University Students, United Kingdom

Technical Appendix

Technical Appendix Table 1. Characteristics of meningococcal carriage in first-year university students, University of Nottingham, UK, 2015–16*

		Genogroup											
		Capsule null locus		Nongroupable†		B		Y		W‡		Non-BYW	
Time point	Carriage rate	No. of isolates (% carried strains)	% of all participants (95% CI)	No. of isolates (% carried strains)	% of all participants (95% CI)	No. of isolates (% carried strains)	% of all participants (95% CI)	No. of isolates (% carried strains)	% of all participants (95% CI)	No. of isolates (% carried strains)	% of all participants (95% CI)	No. of isolates (% carried strains)	% of all participants (95% CI)
SEP	110/769 (14%)	32 (29.1)	4.2 (2.8–5.6)	9 (8.2)	1.2 (0.4–1.9)	25 (22.7)	3.3 (2.0–4.5)	14 (12.7)	1.8 (0.88–2.8)	5 (4.5)	0.7 (0.1–1.2)	25 (22.7)	3.3 (2.0–4.5)
NOV	136/353 (39%)	32 (23.5)	9.1§ (6.1–12.1)	9 (6.6)	2.5 (0.9–4.2)	30 (22.1)	8.5§ (5.6–11.4)	8 (5.9)	2.3 (0.7–3.8)	24 (17.6)	6.8§ (4.2–9.4)	33 (24.3)	9.3§ (6.3–12.4)
MAR	133/288 (46%)	46 (34.6)	16.0§ (11.7–20.2)	5 (3.8)	1.7 (0.2–3.2)	17 (12.8)	5.9 (3.2–8.6)	11 (8.3)	3.8 (1.6–6.0)	23 (17.3)	8.0§ (4.9–11.1)	31 (23.3)	10.8§ (7.2–14.3)

*CI = confidence interval
†Isolates lacking *ctrA*
‡Overall, of the 52 genogroup W isolates, 47 (90%) were serotyof pe 2a and 32 (62%) expressed serogroup W capsule. In September, of the W:2a isolates, 2/4 (50%) expressed serogroup W capsule. In November, of the W:2a isolates, 12/23 (52%) expressed serogroup W capsule. In March, the W:2a isolates, 15/20 (75%) expressed serogroup W capsule.
§Statistically significant difference compared with genogroup-specific carriage rate in September (p<0.001).

Technical Appendix Table 2. MenW carriers in each of 5 dormitories and their respective MenACWY vaccination status, University of Nottingham, UK, 2015–16

		Dormitory											
		A		B		C		D		E		Total	
Time point*	MenACWY vaccination status†	No. (%) of MenW carriers	No. (%) of persons sampled	No. (%) of MenW carriers	No. (%) of persons sampled	No. (%) of MenW carriers	No. (%) of persons sampled	No. (%) of MenW carriers	No. (%) of persons sampled	No. (%) of MenW carriers	No. (%) of persons sampled	No. (%) of MenW carriers	No. (%) of persons sampled
NOV	Vaccinated	3 (50)	45 (69)	4 (80)	50 (78)	2 (50)	52 (68)	3 (75)	43 (57)	4 (80)	67 (93)	16 (67)‡	257 (73)
	Nonvaccinated	3 (50)	18 (28)	1 (20)	12 (19)	2 (50)	24 (31)	1 (25)	31 (41)	1 (20)	5 (7)	8 (33)§	90 (25)
	Unassigned¶	0	2 (3)	0	2 (3)	0	1 (1)	0	1 (1)	0	0	0	6 (2)
	Total	6 (100)	65 (100)	5 (100)	64 (100)	4 (100)	77 (100)	4 (100)	75 (100)	5 (100)	72 (100)	24 (100)	353 (100)
MAR	Vaccinated	9 (100)	50 (79)	3 (75)	43 (65)	4 (80)	57 (77)	3 (100)	24 (65)	2 (100)	39 (81)	21 (91)#	213 (74)
	Nonvaccinated	0	13 (21)	1 (25)	23 (35)	1 (20)	17 (23)	0	13 (35)	0	9 (19)	2 (9)**	75 (26)
	Total	9 (100)	63 (100)	4 (100)	66 (100)	5 (100)	74 (100)	3 (100)	37 (100)	2 (100)	48 (100)	23 (100)	288 (100)

*Students at the September 2015 time point were recruited during registration and are not shown. Of these, 76/769 (10%) were not living in dormitories. The carriage rate for this specific group (9.2% [95% CI = 2.7%–15.7%]) was lower than that for students living in dormitories (14.9% [95% CI = 12.2%–17.5%]), but the difference was not statistically significant ($p > 0.05$).
†Students received MenACWY vaccine before or during registration (September 2015).
‡Of these, 8/16 (50%) expressed serogroup W capsular polysaccharide.
§Of these, 5/8 (63%) expressed serogroup W capsular polysaccharide.
¶Vaccination history unavailable.
#Of these, 15/21 (71%) expressed serogroup W capsular polysaccharide.
**Of these, 1/2 (50%) expressed serogroup W capsular polysaccharide.
